# Critically ill patient – a permanent challenge

**DOI:** 10.1186/1471-2334-14-S7-P91

**Published:** 2014-10-15

**Authors:** Doina Iovănescu, Cleo Roşculeț, Andrei Rogoz, Marius Radu, Cătălin Apostolescu, Raluca Zlotea, Bogdana Manu, Nicuşor Tănase

**Affiliations:** 1 National Institute for Infectious Diseases "Prof. Dr. Matei Balş", Bucharest, Romania

## Background

We present the winding, progressively aggravating evolution of a 70 year-old patient, with multiple risk factors (arterial hypertension, NYHA class II/III heart failure, atrial fibrillation, sequelar stroke, bladder AK, treated with chemotherapy, urinary catheter carrier) who developed sepsis of multiple bacterial etiology – MDR „hospital” Gram-negative bacilli (GNB) and fungi.

## Case report

We present the patient evolution, who in February 2014, following emergency surgery for occlusive syndrome presented severe cardiac decompensation and MDR GNB bronchopneumonia requiring advanced support of vital functions and various combinations of antibiotics (ABT) decided in the ICU. After 32 days of complex therapeutic efforts and support of vital functions (neurological, respiratory, heart, kidney and liver), the patient was extubated but the febrile syndrome recurred, and the biological and imaging data demonstrated a septic evolutionary process. The positive cultures for *P. aeruginosa*, *A. baumannii*, *K. pneumoniae*, all MDR and *Candida* spp., in conjunction with biological and PCQT changes required infectious diseases consultation. The recommend therapy: meropenem + colimycin + linezolid + fluconazole in adequate dose.

The evolution of the infectious process appeared to be controlled without fever after 24h of treatment and the procalcitonin level decreased. Our patient was transferred for further ABT + AFT (antifungal therapy). During hospitalization in the ICU of INBI the patient continued ABT + AFT, slowly evolving favorably and we obtained: evidence of negative microbial cultures, without fever, in the absence of ABT and AFT (for a period of 12 days).

The patient presented a new episode of sepsis caused by *K. pneumoniae* – PDR (carbapenemase positive) with positive urine and blood cultures. Reintroduction of a new „rescue” ABT scheme – meropenem + tigecycline + colimycin, with microbiologic control proving the off- label dosing.

## Conclusion

In the era of microbial resistance we believe that interdisciplinary team decisions are absolutely mandatory from the start, especially in the critically ill patient, requiring associations ABT ± AFT, strictly individualized, often including off-label doses that require specialized expertise. Despite the efforts made and the evidence of microbiological control, as it was likely to happen due to comorbidities and severity of vital organ dysfunction, the patient developed severe heart rhythm disorder followed by exitus. So, in patients with severe immunosuppression, we are continuously forced to maximize therapy regardless of pan-resistance in vitro and trying, at the least, to demonstrate control of the septic process.

## Consent

Written informed consent was obtained from the next of kin for publication of this Case report and any accompanying images. A copy of the written consent is available for review by the Editor of this journal.

